# Systematic Review and Meta-Analysis of Fear of COVID-19

**DOI:** 10.3389/fpsyg.2021.661078

**Published:** 2021-06-11

**Authors:** Faxiang Luo, Reza Ghanei Gheshlagh, Sahar Dalvand, Sholeh Saedmoucheshi, Qingyun Li

**Affiliations:** ^1^Disinfection Supply Center, Yichun People's Hospital, Yichun, China; ^2^Spiritual Health Research Center, Research Institute for Health Development, Kurdistan University of Medical Sciences, Sanandaj, Iran; ^3^Functional Neurosurgery Research Center, Shohada Tajrish Comprehensive Neurosurgical Center of Excellence, Shahid Beheshti University of Medical Sciences, Tehran, Iran; ^4^Department of Epidemiology and Biostatistics, School of Public Health, Tehran University of Medical Sciences, Tehran, Iran; ^5^Salamat Hospital, Kermanshah University of Medical Sciences, Kermanshah, Iran; ^6^Women's Ward of Department of Psychosomatics, Third People's Hospital of Yichun, Yichun, China

**Keywords:** fear, COVID-19, systematic review, meta-analysis, fear of COVID-19

## Abstract

**Background:** Due to lack of preparedness of health systems, fast spread of the new virus, high mortality rates, and lack of a definite treatment, the outbreak of Coronavirus disease (COVID-19) led to high levels of fear and anxiety in different populations. In addition, isolation, mental disorders, and limitations in social interactions as a result of lockdown and travel ban increased the fear of the new coronavirus.

**Methods:** International databases, including Scopus, PubMed, Web of Science, and Google scholar, were searched without any time limitation, and all observational studies published in English reporting the mean of fear of COVID-19 based on the Fear of COVID-19 scale (FCV-19S) were included in the analysis. Methodological quality was assessed using the Strengthening the Reporting of Observational Studies in Epidemiology (STROBE) guidelines. Random effects model, subgroup analysis, and meta-regression analysis were used to analyze the data. Heterogeneity across studies was examined using Cochran's *Q* test and *I*^2^ statistic. All the statistical analyses were conducted using R software v4.0.3.

**Results:** A total of 44 articles with a sample size of 52,462 were reviewed. A pooled mean of 18.57 was found for fear of COVID-19. The mean of fear of COVID-19 was higher in women than in men (20.67 vs. 18.21). The highest and lowest means of fear of COVID-19 had been found in Asia (18.36) and Australia (17.43) based on continent, and in hospital staff (19.51) and college students (17.95) based on target population, respectively. In addition, the highest and lowest means of fear of COVID-19 were related to items #1 and #3 of the scale, respectively. According to the results of meta-regression analysis, there was no significant association between the mean of fear of COVID-19 and sample size and participants' age. In addition, publication error was not significant (*P* = 0.721).

**Conclusion:** The mean of fear of COVID-19 was high around the world; therefore, it seems necessary to pay more attention to the negative effects of the COVID-19 pandemic on mental health.

## Introduction

The Coronavirus disease (COVID-19) pandemic was first reported in Hubei, China, in December 2019. So far, it has affected about six million people and has led to the death of more than 360,000 people around the world mostly due to severe acute respiratory illness (Ashamalla et al., 2020). Given the lack of an effective treatment for COVID-19, different countries around the world focused their efforts on reducing the risk of transmission through implementing public health measures, such as social distancing, self-isolation, and regular hand washing. In addition, unprecedented measures, such as controlling borders, contact tracing, and lockdown were taken (Ahorsu et al., 2020a; Alyami et al., 2020). These measures led to widespread fear so that in some countries people started to stockpile staple foods, toilet paper, and even guns (Bakioğlu et al., 2020; Skoda et al., 2020). As the prevalence of COVID-19 increased, people started to isolate themselves, limit their social interactions, and avoid others for fear of getting the virus (Abuhammad et al., 2020). Fear is an adaptive response to one's environment and a defense mechanism to increase the chance of one's survival; however, it can be maladaptive when it is not proportionate to the actual threat (Steimer, 2002).

During the Severe Acute Respiratory Syndrome (SARS) and Ebola outbreaks, public fear worsened the negative effects of the actual illness; therefore, one of the most important challenges in the face of outbreaks is to control social reactions (García-Reyna et al., 2020). In order to reduce possible psychological problems, researchers recommend that the level of fear, worry, and helplessness associated with COVID-19 should be examined, because high levels of stress may prevent one from making logical decisions to protect themselves (Ahorsu et al., 2020a). For example, some patients who need medical care may refuse to go to the hospital due to experiencing illogical levels of fear (WongLaura et al., 2020). Some patients may postpone their surgical treatment for fear of contracting the virus (Vanni et al., 2020). In some cases, fear of COVID-19 can lead to hypochondriasis, so that some people may misinterpret their bodily sensations and attribute them to COVID-19 (Coelho et al., 2020). Some people may also excessively use medications recommended in COVID-19 treatment guidelines, such as hydroxychloroquine (Banerjee, 2020). On the other hand, fear can act as a motivator of behavioral change in the face of COVID-19 (Harper et al., 2020; Pakpour and Griffiths, 2020). The experience of fear can increase risk perception and reinforce protective behaviors, such as washing of hands and keeping physical distance (Broche-Pérez et al., 2020). When people take a threat seriously, they can perform preventive measures more efficiently, and perception of threat as a motivator facilitates the prevention of COVID-19. Harper et al. found that fear of COVID-19 strongly predicted improved social distancing and hand washing and had an important role in adherence to public health measures related to COVID-19 (Harper et al., 2020).

The COVID-19 pandemic has led to fear and negative emotions; however, it has also had positive consequences, such as encouraging people to engage in ethical behavior (Jian et al., 2020). According to what was explained above, measuring fear of COVID-19 has an important role in understating the implications of the pandemic for mental health and in designing interventions to reduce COVID-19 fear and anxiety. One of the most important instruments available to assess fear of COVID-19 is the Fear of COVID-19 Scale (FCV-19S) that has been translated to many languages. Studies form different parts of the world examining fear of COVID-19 have led to different results. Therefore, the goal of the present systematic review and meta-analysis is to estimate the pooled mean of fear of COVID-19 around the world.

## Methods

The present systematic review and meta-analysis aimed to estimate the pooled mean of fear of COVID-19 based on the Preferred Reporting Items for Systematic Reviews and Meta-Analyses (PRISMA) guidelines (Moher et al., 2009).

### Data Sources and Search Strategy

Search for articles was conducted in September 20, 2020, in databases of Web of science/ISI, PubMed, and Scopus using the following keywords: Wuhan Coronavirus, Sars-cov-2, 2019 Novel Coronavirus, COVID-19 Virus, Coronavirus Disease 2019 Virus, Wuhan Seafood Market Pneumonia Virus, Fear, and all possible combination to increase search sensitivity. In addition, references of the selected articles were reviewed to access more related articles.

### Selection Criteria

All observational studies published in English examining the state of fear of COVID-19 using the FCV-19S were analyzed. This scale developed by Ahorsu et al. assesses fear of COVID-19 using seven items that are rated on a Likert-type scale ranging from 5 (totally agree) to 1 (totally disagree). Total score on this scale ranges from 7 to 35, and higher scores indicate stronger fear of COVID-19 (Ahorsu et al., 2020a). The inclusion criteria were as follows: participants aged at least 18 years and reporting the mean and standard deviation of fear of COVID-19 score. Articles with unavailable full texts, preprinted articles, and articles not reporting the fear of COVID-19 score were excluded from the analysis.

### Data Collection

In the first step, two independent authors screened the articles and selected those having the aforementioned keywords in their titles or abstracts. Then, they extracted the article information and recorded it in a predesigned Excel sheet. This information included first author's name, publication year, mean age of patients, target population, mean and standard deviations of fear of COVID-19 (total score and score by gender). Because all the selected articles had been published in 2020, publication year was not included in the table presenting article information.

### Quality Assessment

The two researchers independently evaluated the quality of the articles based on 10 items of Strengthening the Reporting of Observational Studies in Epidemiology (STROBE) checklist (title and abstract, study environment, objectives and hypotheses, sample size, inclusion criteria, statistical methods, descriptive data, interpretation of findings, limitations, and funding). Higher scores indicate better methodological quality. In terms of methodological quality, articles were divided into three categories: poor (4 or below), moderate (4 to 7), and good (over 7) (Vandenbroucke et al., 2007).

### Analysis

Random effects model was used to estimate the pooled raw mean of fear of COVID-19. A forest plot was used to visually depict heterogeneity across studies in which the mean of fear of COVID-19 with a 95% confidence interval and also the pooled raw mean of the selected studies are reported. Heterogeneity across studies was assessed using *I*^2^ statistic and Cochran's *Q* test. *I*^2^ percentages of 25%, 50%, and 75% show low, average, and high heterogeneity, respectively, and in Cochran's *Q* test, *P* < 0.1 indicates significant heterogeneity (Higgins et al., 2003). Source of heterogeneity across studies was examined using subgroup analysis by gender, continent (Asia/America/Europe/Australia/multi-countries), and target population (general population, college students, pregnant women, and medical staff).

The association of the mean of fear of COVID-19 with sample size and mean age of participants was assessed using meta-regression analysis. To ensure that the meta-regression results were not affected by one or several articles, leave-one-out sensitivity analysis method was used that involved performing the analysis on the data by leaving out one study at a time. For studies reporting scores by drop out one study at a time and estimate the pooled raw mean of remained studies. In addition, the potential effect of small studies was assessed using a funnel plot based on Egger's regression test (Egger et al., 1997). It is worthwhile to note that all analyses were performed based on random effects model using R software, version 4.0.3, and that all statistical tests were two-tailed ones. In addition, the significance level for all tests except for examination of heterogeneity across studies was set at *P* < 0.05.

## Results

**Figure 1:**
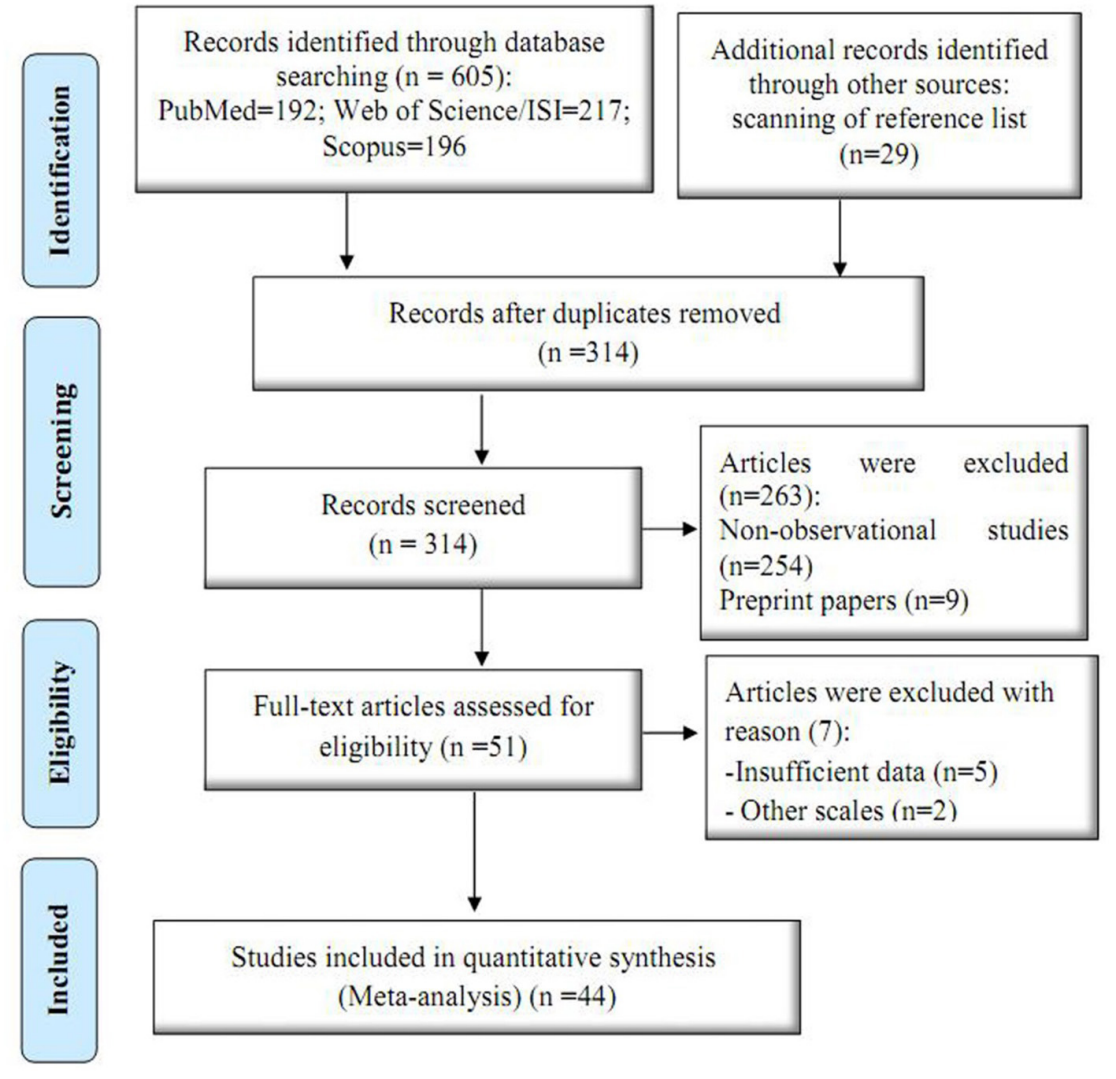
Process of selecting and screening articles.

In the primary search, a total of 634 articles were found, of which 320 duplicate articles were excluded, and titles and abstracts of the remaining articles were reviewed. In addition, 270 articles focused on unrelated subjects were removed from the analysis. Figure 1 shows the flowchart of selecting and screening articles based on the PRISMA guidelines.

A total of 44 articles with a sample size of 52,462 were included in the final analysis. As shown in Table 1, among the 44 articles, 33 reported the total score of fear of COVID-19, 8 reported this score by gender, and 17 reported the mean scores by item (Abuhammad et al., 2020; Alyami et al., 2020; Bitan et al., 2020; Cavalheiro and Sticca, 2020; Caycho-Rodríguez et al., 2020; Elemo et al., 2020; Giordani et al., 2020; Huarcaya-Victoria et al., 2020; Martínez-Lorca et al., 2020; Masuyama et al., 2020; Pang et al., 2020; Sakib et al., 2020; Satici et al., 2020a; Soraci et al., 2020; Winter et al., 2020; Zolotov et al., 2020). There were two groups of participants in the Winter et al. study; therefore, results were reported separately for each group. In eight studies, the mean of fear of COVID-19 was reported by gender (Abad et al., 2020; Bakioğlu et al., 2020; Broche-Pérez et al., 2020; Haktanir et al., 2020; Hossain et al., 2020; Mertens et al., 2020; Nguyen et al., 2020a; Sakib et al., 2020). These studies had been conducted with the general population, college students, pregnant women, and medical staff. Detailed information on selection of articles is provided in Table 1. In terms of methodological quality, eight studies had medium quality, and the remaining articles had excellent quality.

**Table 1:** The characteristics of selected paper.

**References**	**Sample size (M)**			**Age**	**Place**	**FCV-19S mean score**			**Target population**
	**Total**	**Male**	**Female**			**All**	**Male**	**Female**	
Giordani et al. (2020)	7,430	1903	5527		Brazil	19.8 ± 5.3	-	-	General population
Winter et al. (2020)	1,397	-	-	-	New Zealand	15.6 ± 7.7	-	-	General population
	1,023	-	-	-	New Zealand	18.3 ± 7.9	-	-	General population
Lin et al. (2020)	1,078	628	450	26.2 ± 7.4	Iran	1,028 ± 4.45	-	-	General population
Haktanir et al. (2020)	668	187	481	29.3 ± 10.7	Turkey	-	16.99 ± 5.15	19.06 ± 5.42	General population
Seyed Hashemi et al. (2020)	651	245	406	33.5 ± 10.8	Iran	18.72 ± 5.81	-	-	General population
Saricali et al. (2020)	786	224	562	24 ± 7.8	Turkey	17.76 ± 6.01	-	-	General population
Saravanan et al. (2020)	433	278	155	21 ± 2.9	United Arab Emirates	16.6 ± 6.3	-	-	University students
Salehi et al. (2020)	222	0	222	29.1 ± 5.6	Iran	22.5 ± 5.9	-	-	Pregnant women
Rodríguez-Hidalgo et al. (2020)	640	179	461	21.6 ± 4	Spain	14.37 ± 5.38	-	-	University students
Nguyen et al. (2020a)	5,423	2,602	2,821	22 ± 2	Vietnam	16.7 ± 5.3	16.2 ± 5.6	17 ± 4.8	University students
Mertens et al. (2020)	439	132	307	-	Multiple countries	25.85 ± 5.91	25.05 ± 6.28	26.16 ± 5.73	General population
Rahman et al. (2020)	587	224	363	41.3 ± 12.5	Australia	18.4 ± 6.5	-	-	General population
Martínez-Lorca et al. (2020)	606	109	497	21.6 ± 3	Spain	16.79 ± 6.04	-	-	University students
Labrague and de Los Santos (2020)	261	69	192	30.95	Philippines	19.92 ± 6.15	-	-	Hospital staff
Konstantinov et al. (2020)	466	154	312	19 ± 2.7	Kazakhstan	22.1 ± 5.8	-	-	University students
Kaya et al. (2020)	1,012	185	827	28.3 ± 8.7	Turkey	19.1 ± 6.3	-	-	General population
Jaspal et al. (2020)	411	-	-	48.85 ± 15.38	United Kingdom	25.67 ± 7.55	-	-	General population
Gasparro et al. (2020)	735	195	240	44.8 ± 12.4	Italy	15.03 ± 5.45	-	-	Dentists
Cavalheiro and Sticca (2020)	354	163	191	34.9 + 7.3	Brazil	15.76 ± 6.21	-	-	General population
Broche-Pérez et al. (2020)	772	203	569	34 + 14.6	Cuba	-	17.9 ± 80.5	21.9 ± 6.9	General population
García-Reyna et al. (2020)	2,860	1,219	1,641	35.4 ± 8	Mexico	19.3 ± 6.9	-	-	Hospital staff
Abuhammad et al. (2020)	1,655	598	1,057	29.5 ± 7.7	Jordan	21.80 ± 6.43	-	-	General population
Ahorsu et al. (2020b)	413	256	157	57.7 ± 7.3	Iran	21.80 ± 6.43	-	-	General population
Ahorsu et al. (2020c)	580	290	290	-	Iran	15.90 ± 5.29	-	-	Pregnant women
Sakib et al. (2020)	8,550	4,790	3,760	26.5 ± 9	Bangladesh	**-**	20.29 ± 5.90	22.75 ± 5.65	General population
Reznik et al. (2020)	547	-	-	-	Russia	17.4 ± 4.7	-	-	University students
Reznik et al. (2020)	276	-	-	-	Belarus	16.6 ± 4.5	-	-	University students
Zolotov et al. (2020)	370	77	289	25.2 ± 3.1	Israel	14.95 ± 4.80	-	-	University students
Abad et al. (2020)	1,844	368	1,468	36.2	Brazil	18.1 ± 6.7	14.5 ± 0.3	18.9 ± 6.6	General population
Perz et al. (2020)	237	64	173	30.3 ± 10.2	USA	18.1 ± 7.1	-	-	University students
Yehudai et al. (2020)	291	49	242	24.5 ± 5.5	Israel + Russia	22 ± 6.3	-	‘-	University students
Masuyama et al. (2020)	629	302	327	12.9 ± 0.83	Japan	18.71 ± 5.65	-	-	University students
Hossain et al. (2020)	2,157	1,166	991	33.4 ± 14.6	Bangladesh	18.53 ± 5.01	18.07 ± 4.94	19.07 ± 5.04	General population
Isralowitz et al. (2020)	598	173	425	-	Multiple countries	21.2 ± 6.1	-	-	University students
Bakioğlu et al. (2020)	960	297	663	29.7 ± 9.6	Turkey	19.44 ± 6.07	16.82 ± 5.75	20.61± 5.85	General population
Alyami et al. (2020)	639	370	269	34.7 ± 11.8	Saudi Arabia	-	-	-	General population
Satici et al. (2020a)	1,304	387	917	29.4 ± 10.5	Turkey	-	-	-	General population
Bitan et al. (2020)	339	97	240	-	Israel	-	-	-	General population
Caycho-Rodríguez et al. (2020)	1,291	268	1,023	39.3 ± 15.7	Argentine	-	-	-	General population
Elemo et al. (2020)	307	249	58	30.9 ± 7.9	Ethiopia	-	-	-	General population
Huarcaya-Victoria et al. (2020)	832	286	546	38.3 ± 12.7	Peru	-	-	-	General population
Pang et al. (2020)	228	66	162	26	Malaysia	-	-		General population
Soraci et al. (2020)	249	20	229	34.5 ± 12.2	Italy	-	-		General population

*FCV-19S, The Fear of Coronavirus-19 Scale*.

**Figure 2:**
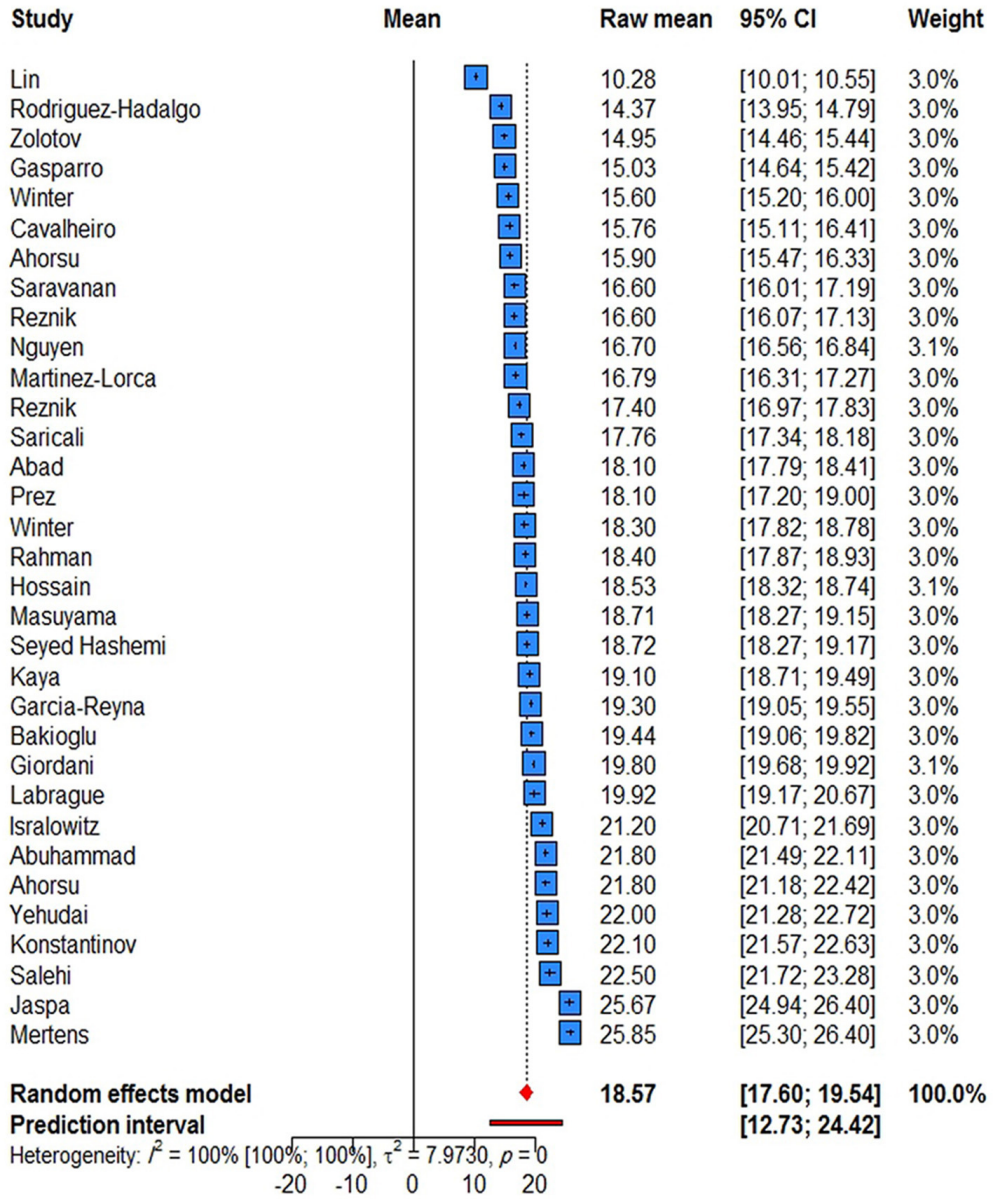
Forest plot showing the pooled mean of fear of COVID-19 (*N* = 33).

According to the level of heterogeneity across studies, random effects model was used to combine the studies (*P* < 0.0001; *Q* = 8243.69, df = 32, *P* < 0.0001, τ^2^ = 7.9730, and *I*^2^= 99.6%). In the present study, the pooled mean of fear of COVID-19 was found to be 18.57 (95% CI: 17.60–19.54). In addition, the prediction interval for the pooled mean of fear of COVID-19 was found to be 12.72–24.42 (Figure 2). Among the selected studies, 17 reported the mean of fear of COVID-19 by item; the highest and lowest mean scores were related to items #1 (3.32) and #3 (1.78), respectively. It is worthwhile to note that the mean of fear of COVD-19 for all items was higher in Asian studies compared to those conducted in other continents. The lowest scores on items #1, #3, #5, and #6 were reported by the American studies, the lowest scores on items #4 and #7 were reported by Australian studies, and the lowest scores on item #2 were reported by European studies. In addition, the mean of fear of COVD-19 on all items except for items #1 and #4 was higher in the general population than in college students. The pooled mean of fear of COVD-19 by item is presented in Table 2 (Supplementary Figures 1–7).

**Table 2:** Pooled mean of fear of COVD-19 by item.

**No**.	**Item**	**Mean**	**95% CI**
1	I am most afraid of coronavirus-19.	3.32	3.16–3.48
2	It makes me uncomfortable to think about coronavirus-19.	3.23	3.10–3.37
3	My hands become clammy when I think about coronavirus-19	1.78	1.57–2.00
4	I am afraid of losing my life because of coronavirus-19	2.64	2.37–2.88
5	When watching news and stories about coronavirus-19 on social media, I become nervous or anxious.	2.97	2.78–3.16
6	I cannot sleep because I'm worrying about getting coronavirus-19.	1.83	1.63–2.02
7	My heart races or palpitates when I think about getting coronavirus-19.	2.02	1.77–2.27

### Subgroup Analysis

The pooled raw mean of fear of COVID-19 was examined using random effects model. According to the results of subgroup analysis, the highest and lowest pooled means of fear of COVID-19 were reported in the studies conducted in Asia (18.36, 95% CI: 16.88–19.84) and Australia (17.43, 95% CI: 15.51–19.34), respectively (Supplementary Figure 8). In addition, the pooled raw mean of fear of COVID-19 was higher in medical staff than in other groups (Supplementary Figure 9).

According to the results of subgroup difference test reported in Table 3, there was a significant difference in the mean of fear of COVID-19 in different continents (*P* = 0.0347), but there was no significant difference between different target populations (*P* = 0.0773). In addition, eight articles reported the mean of fear of COVID-19 by gender that was lower in men (18.21, 95% CI: 15.99–20.42) than in women (20.67, 95% CI: 18.62–22.73) (Supplementary Figures 10, 11). Moreover, the mean fear of COVID-19 was 17.68 and 19.70 in Asian men and women and 16.15 and 20.39 in American men and women, respectively (Supplementary Figures 12, 13).

**Table 3:** Subgroup analysis of the pooled mean of fear of COVID-19 by continent and target population.

**Group**		**No. S**	**Pooled mean**	**Confidence level (95%)**	**Heterogeneity**	**Subgroup differences test**		
					***I*^**2**^ (%)**	***Q***	**df**	***P***
Continent	Asia	17	18.36	16.88–19.84	99.7	10.36	4	0.0347
	America	5	18.25	17.22–19.28	98.3			
	Europe	5	17.68	14.68–20.68	99.5			
	Australia	3	17.43	15.51–19.34	98			
	Multi countries	3	23.02	20.01–26.02	98.8			
Target group	General population	16	19.05	17.42–20.69	99.8	5.12	2	0.0773
	University student	12	17.95	16.37–19.18	99			
	Hospital staff	2	19.51	19.93–20.08	58			

**Figure 3:**
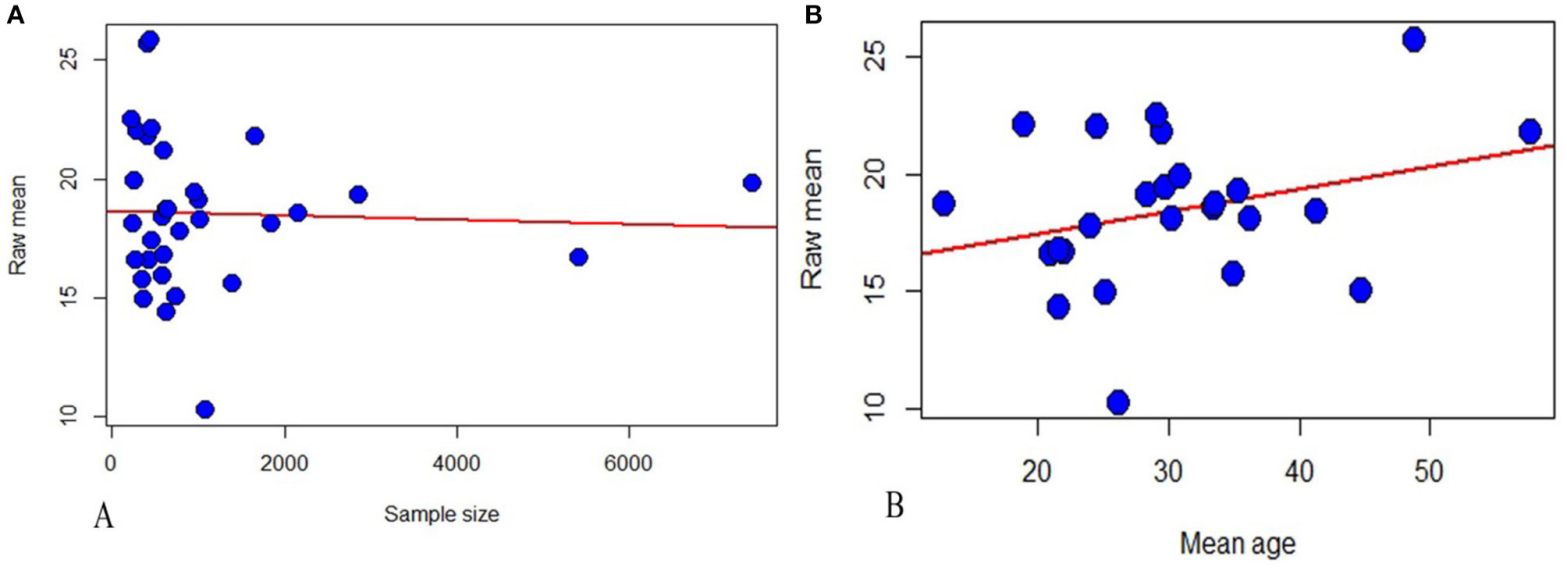
Meta-regression analysis of the relationship between mean of fear of COVID-19 and sample size **(A)** and mean age of participants **(B)**.

The results of meta-regression analysis showed that mean score of fear of COVID-19 increased with mean age, but the relationship was not statistically significant (*P* = 0.797) (Figure 3 and Table 4).

**Table 4:** Univariate meta-regression analysis.

**Variable**	**Estimate**	**Standard error**	***P*-value**
Sample size	−0.0001	0.0004	0.7972
Mean age	0.0956	0.0613	0.1190

Results of sensitivity analysis based on random effects model showed that none of the studies alone had a substantial impact on the pooled raw mean of fear of COVID-19 (Supplementary Figure 14). Results of Egger's regression test also indicated that publication bias was not statistically significant (*P* = 0.721) (Supplementary Figure 15).

Examination of the pooled mean by continent and target population showed that on all items, it was higher in the studies conducted in Asia compared to those conducted in other continents. In addition, the pooled mean of fear of COVID-19 on all items except for items #1 and #4 was higher in the general population than in college students (Table 5). Moreover, a significant difference was observed between studies conducted in different continents in terms of scores on item #2 of the scale (*P* < 0.0001), but there was no significant difference between different continents in terms of scores on other items. In addition, a significant difference was observed between different target populations in scores on items #6 and #7 (*P* < 0.05).

**Table 5:** Subgroup analysis of the mean of different items of the FCV-19s by continent and target population.

**Question**	**Group**	**Subgroup**	**No. S**	**Pooled mean**	**Confidence level (95%)**	**Subgroup differences test**		
						***Q***	**df**	***P***
Q1	Continent	Asia	8	3.41	3.22–3.60	2.32	3	0.509
		America	4	2.98	2.38–3.57			
		Europe	2	3.17	2.65–3.69			
		Australia	2	3.34	2.79–3.89			
	Target	GP	14	3.15	3.15–3.48	0.01	1	0.931
		US	3	3.35	2.65–4.05			
Q2	Continent	Asia	8	3.35	3.24–3.47	28.6	3	<0.0001
		America	4	2.98	2.46–3.49			
		Europe	2	2.97	2.88–3.05			
		Australia	2	3.16	2.61–3.72			
	Target	GP	14	3.26	3.11–3.40	0.83	1	0.363
		US	3	3.11	2.84–3.38			
Q3	Continent	Asia	8	1.89	1.51–2.28	1.67	3	0.643
		America	4	1.59	1.21–1.97			
		Europe	2	1.62	1.39–1.86			
		Australia	2	1.65	1.49–1.81			
	Target	GP	14	1.83	1.60–2.07	2.46	1	0.117
		US	3	1.56	1.31–1.80			
Q4	Continent	Asia	8	2.80	2.43–3.16	5.09	3	0.166
		America	4	2.49	1.62–3.52			
		Europe	2	2.42	2.33–2.50			
		Australia	2	2.29	2.03–2.54			
	Target	GP	14	2.62	2.37–2.87	0.01	1	0.965
		US	3	2.65	1.35–3.95			
Q5	Continent	Asia	8	3.11	2.88–3.33	2.66	3	0.447
		America	4	2.65	2.05–3.25			
		Europe	2	2.95	2.86–3.04			
		Australia	2	2.98	2.35–3.61			
	Target	GP	14	2.99	2.78–3.20	0.91	1	0.339
		US	3	2.88	2.79–2.97			
Q6	Continent	Asia	8	1.91	1.57–2.25	1.31	3	0.720
		America	4	1.66	1.29–2.04			
		Europe	2	1.69	1.44–1.93			
		Australia	2	1.77	1.53–2.00			
	Target	GP	14	1.88	1.67–2.10	4.01	1	0.0453
		US	3	1.56	1.33–1.80			
Q7	Continent	Asia	8	2.12	1.70–2.54	4.96	3	0.175
		America	4	1.82	1.41–2.23			
		Europe	2	2.02	1.91–2.13			
		Australia	2	1.74	1.48–1.99			
	Target	GP	14	2.09	1.82–2.36	3.86	1	0.049
		US	3	1.70	1.43–1.98			

*Nos, Number of studies; GP, General Population, US, University Students*.

## Discussion

The present systematic review and meta-analysis aimed to estimate the pooled mean of fear of COVID-19. The results showed that the participants in the reviewed studies had obtained 41% of the total score on the the FCV-19s. Fear of COVID-19 leads to stigmatization and social exclusion of patients and their families, and makes them vulnerable to adjustment problems, depression, irritability, anxiety, and anger (Abad et al., 2020; Satici et al., 2020b; Zhang et al., 2020). Therefore, it is important to pay attention to implications of COVID-19 for psychological health, because pandemics can lead to crisis in psychological, social, and economic domains (Xiang et al., 2020). Fear is not limited to the COVID-19 pandemic and has been observed in other outbreaks, including those of HIV and SARS (Ho et al., 2005). The pooled mean of fear of COVID-19 was higher in women than in men. This finding can be attributed to the fact that women are more delicate and vulnerable than men. In addition, Bakioğlu et al. (2020) found that it was more acceptable for women to express their fears of illness. On the other hand, it is more acceptable for men to be strong and brave. In addition, because men are less likely than women to become ill, they tend to be less afraid of COVID-19. The results of the present study showed that the highest and lowest fears of COVID-19 scores were in studies conducted in Asia and Australia, respectively. This finding can be attributed to the fact that before spreading to other countries, COVID-19 was reported in China as an Asian country; therefore, people in China and other Asian countries experienced higher levels of fear of the new virus. Overall, different rates of fear of COVID-19 in different countries can be attributed to contextual and cultural factors and different levels of access to medical services. Isolation as a result of the COVID-19 pandemic led to increased rates of mental problems, such as anxiety, anger, PTSD, confusion, and even suicide (Giordani et al., 2020; Haktanir et al., 2020; Mamun and Griffiths, 2020a). For example, a Bangladeshi man killed himself because he thought that he had the new virus, but the autopsy showed that he actually did not (Mamun and Griffiths, 2020b). Therefore, misconceptions about COVID-19 can lead to increased xenophobia and suicide ideation. The same pattern had been observed during the SARS outbreak in Asia (Hong Kong) (Cheung et al., 2008).

The pooled mean of fear of COVID-19 was higher in medical staff than in other target groups. This group became involved in fighting the new virus when health systems were not adequately prepared to respond to the pandemic (1). Long-term exposure to confirmed and also unrecognized COVID-19 patients, insufficient training on prevention and control of infectious diseases, and shortage of protective equipment were factors putting healthcare providers at higher risk of COVID-19 and, as a result, fear of the pandemic (Wang et al., 2020; Zhou et al., 2020). Nguyen et al. showed that the risk of testing positive for COVID-19 was three times higher in healthcare workers than in the general population (Nguyen et al., 2020b). Fear in healthcare providers is not limited to COVID-19 and has been reported during other outbreaks, including those of HIV (Montgomery and Lewis, 1995) and SARS (Ho et al., 2005). The highest and lowest pooled means were related to items #1 and #3 of the scale, respectively. This finding can be attributed to what the items assess. Item #1 directly assesses fear of COVID-19, while item #3 asks about symptoms of fear of COVID-19. One of the limitations of this study was the exclusion of preprint studies. Due to the large number of studies, it was not possible to review this type of articles in this meta-analysis and it is suggested that this type of articles be reviewed in future studies.

## Conclusion

The excessive fear observed in previous outbreaks, including those of HIV and Ebola, has also been reported in the current COVID-19 pandemic and can be observed in the future. Excessive fear can negatively impact one's life in personal (e.g., mental health problems) and social (panic shopping and xenophobia) domains, while a normal (logical) level of fear can help one pay more attention to government measures aimed at reducing the spread of COVID-19 (33). The results of the present study showed that a moderate level of fear is required to reduce the risk of contracting COVID-19 and that fear of COVID-19 can be controlled and prevented from turning into excessive fear through providing effective training programs for different populations.

## Data Availability Statement

The original contributions presented in the study are included in the article/Supplementary Material, further inquiries can be directed to the Corresponding author.

## Author Contributions

FL and RGG: data collection and manuscript preparation. RGG: manuscript preparation and study conceptualization. QL: study design. SS and RGG: search strategy. QL and FL: final revision and grammar editing. SD: statistical analysis.

## Conflict of Interest

The authors declare that the research was conducted in the absence of any commercial or financial relationships that could be construed as a potential conflict of interest.
