# Supplementary material for: Systematic Review and Meta-Analysis of Fear of COVID-19
Source: Front Psychol. 2021 Jun 11;12:661078. doi: 10.3389/fpsyg.2021.661078 (PMC8231929; doi:10.3389/fpsyg.2021.661078)
Supplement: Supplementary file 1 [file Data_Sheet_1.docx]

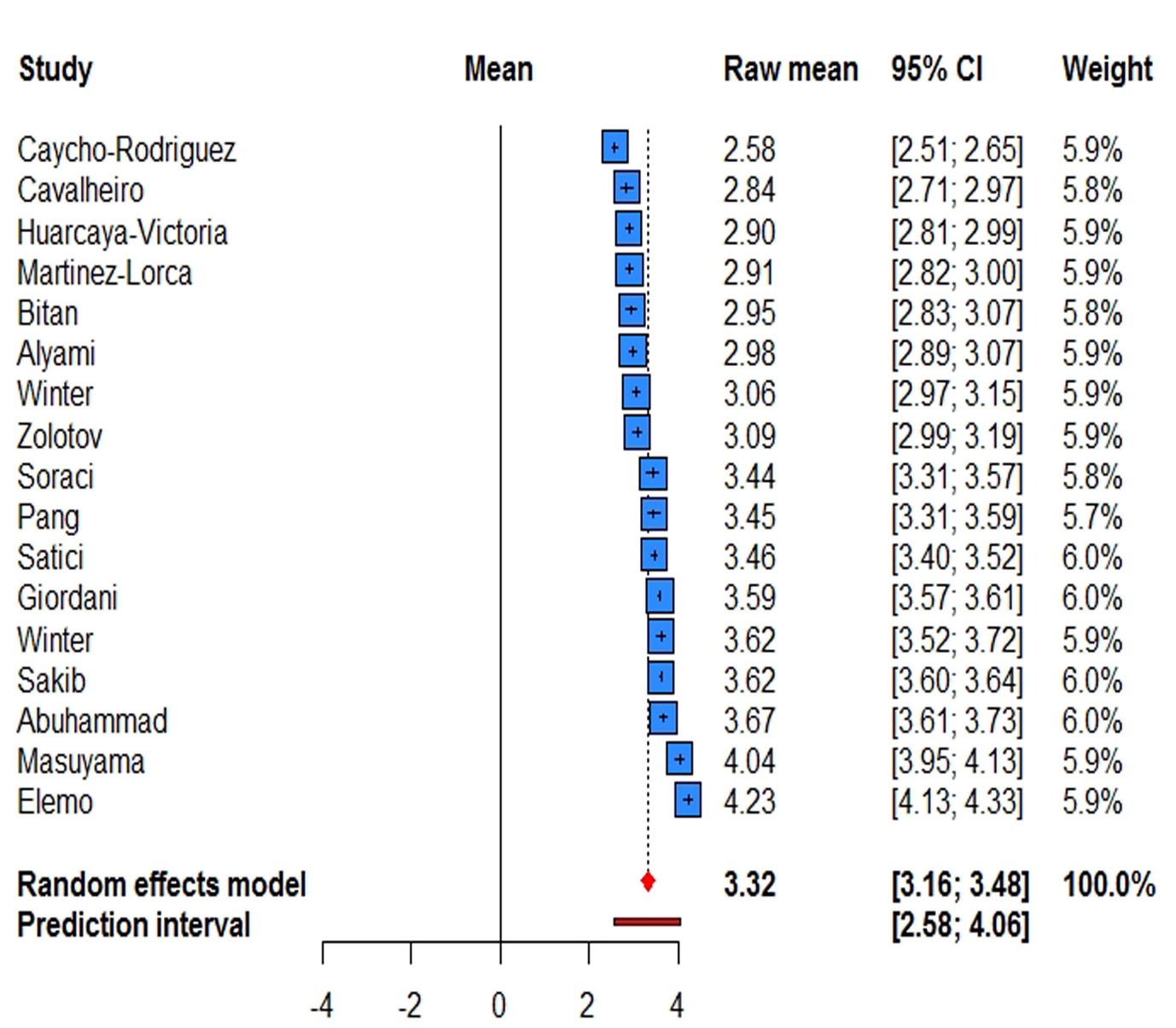


**Supplementary figure 1:** Forest plot of the fear Score mean of question 1

**
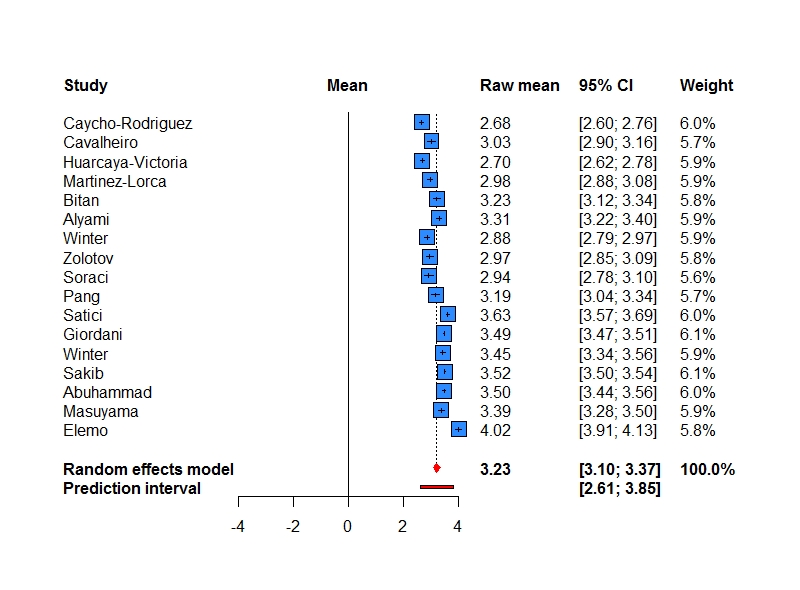
**

**Supplementary figure 2:** Forest plot of the fear Score mean of question 2


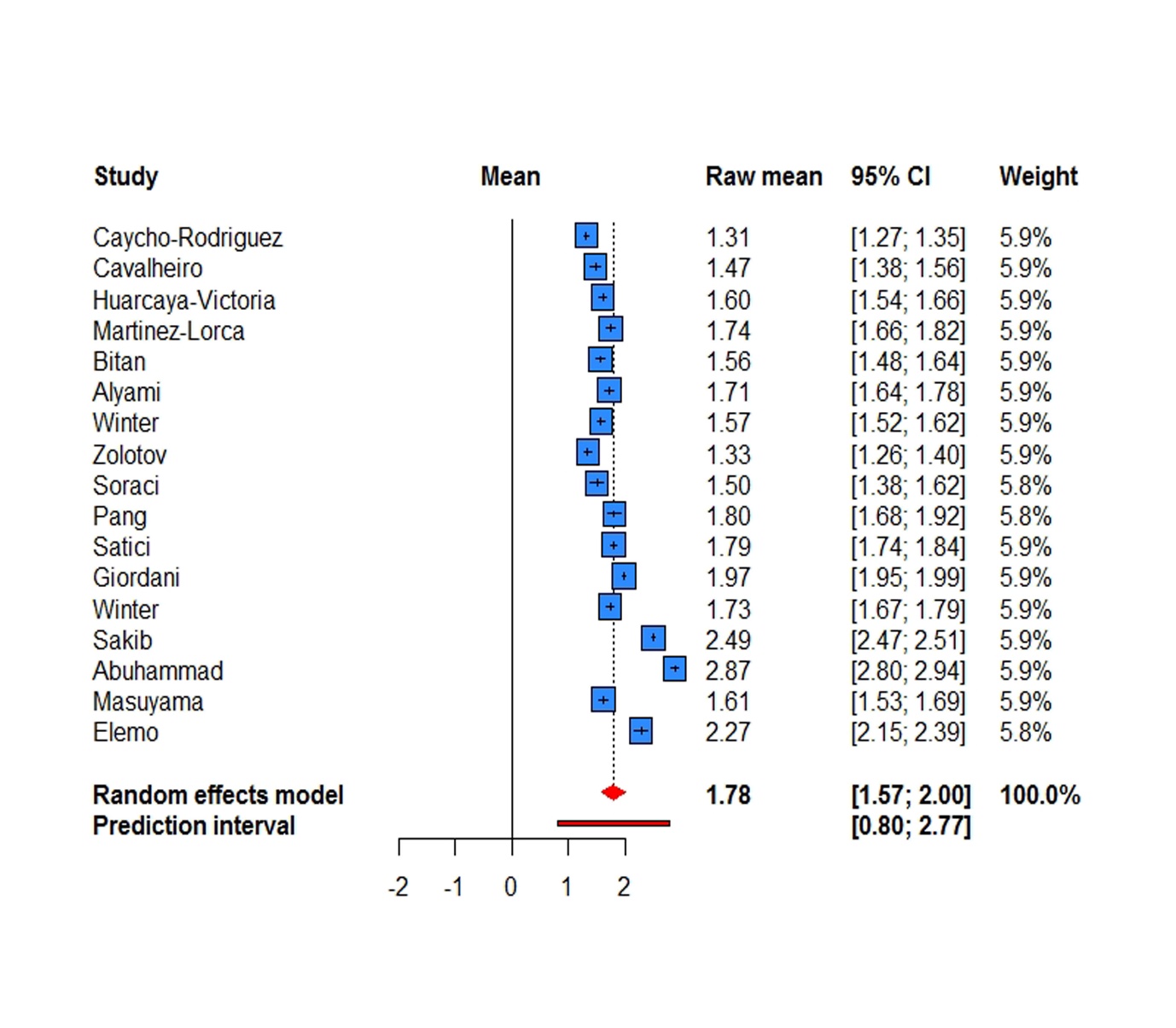


**Supplementary figure 3:** Forest plot of the fear Score mean of question 3

**
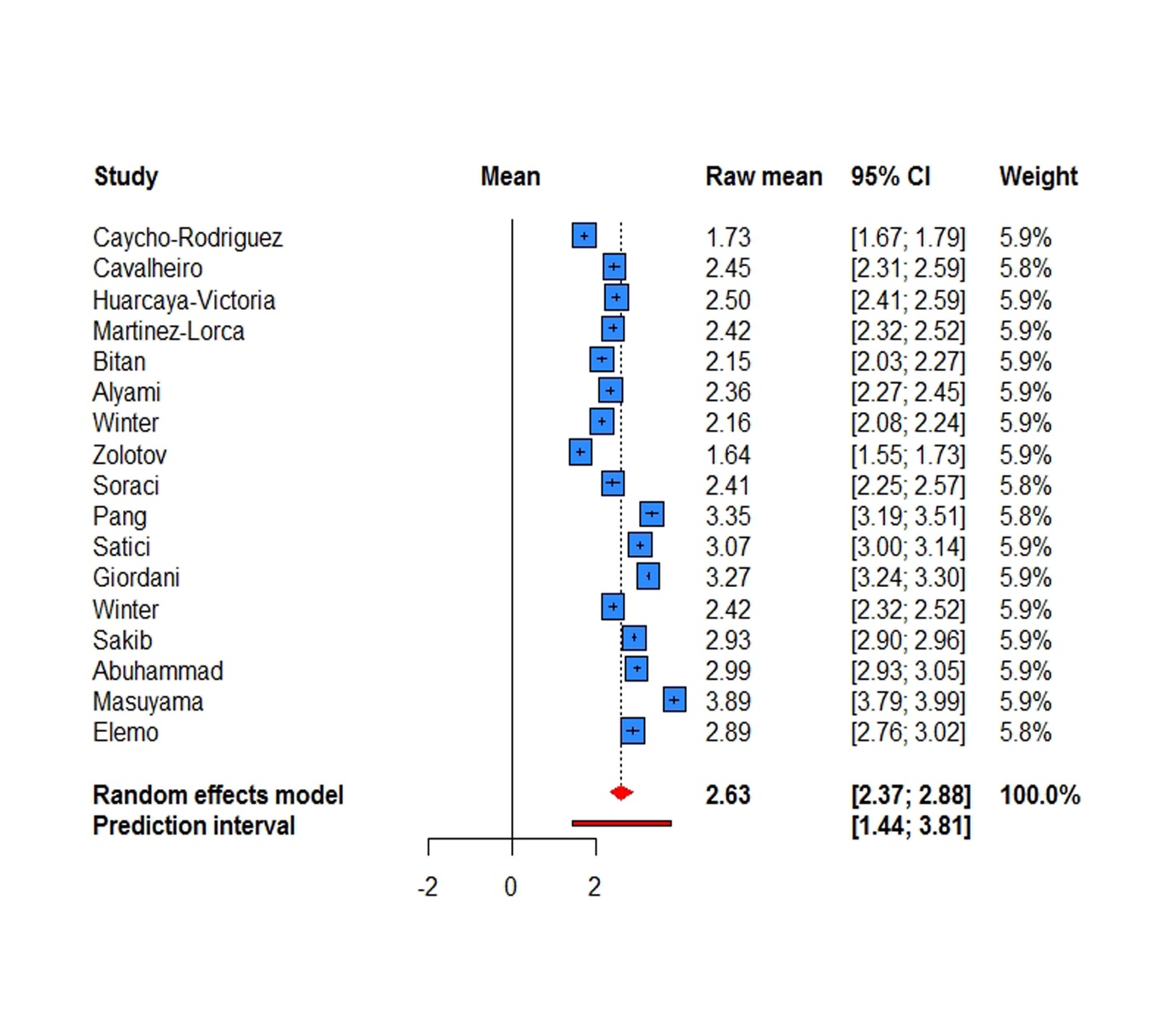
**

**Supplementary figure 4:** Forest plot of the fear Score mean of question 4


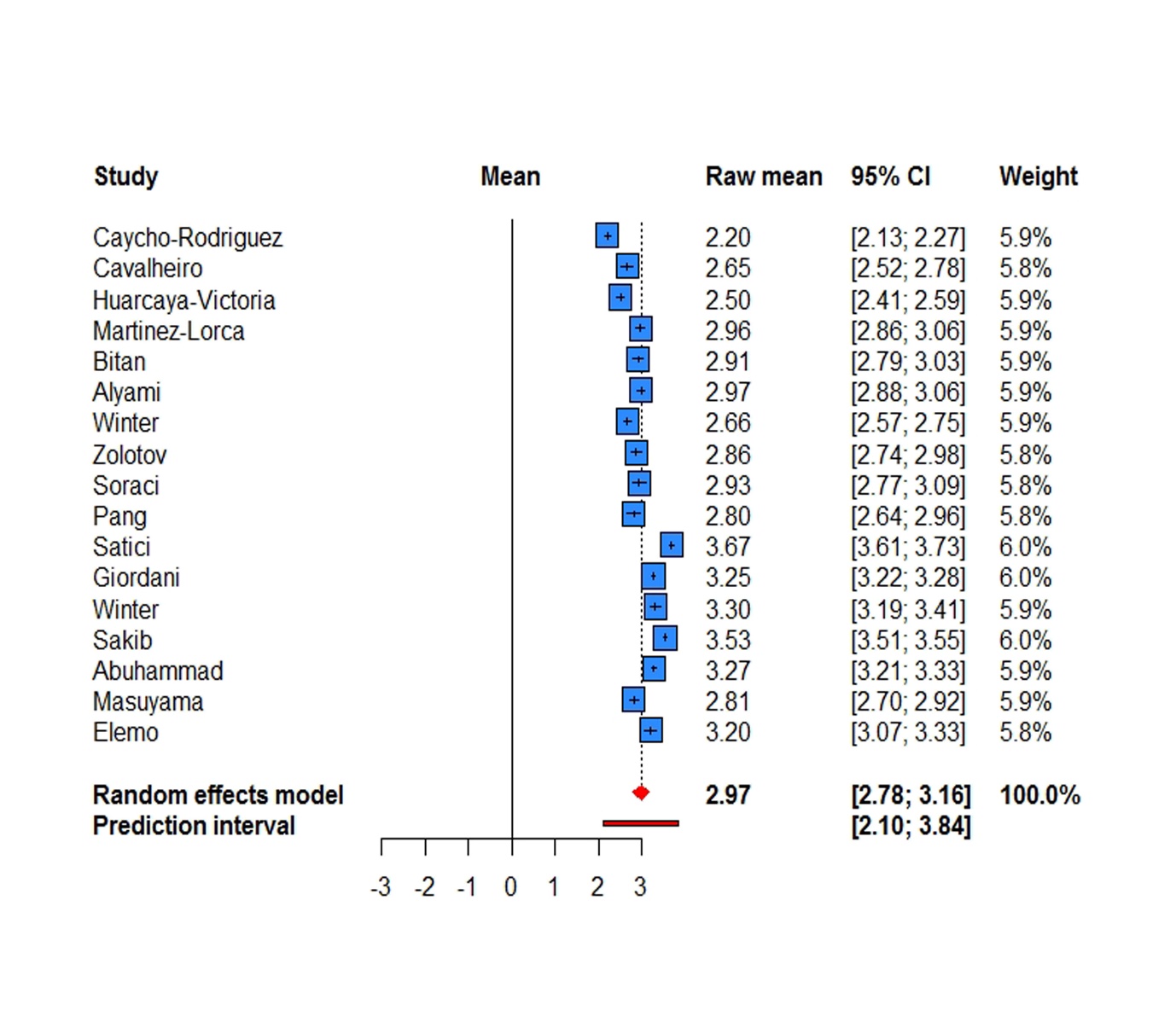


**Supplementary figure 5:** Forest plot of the fear Score mean of question 5


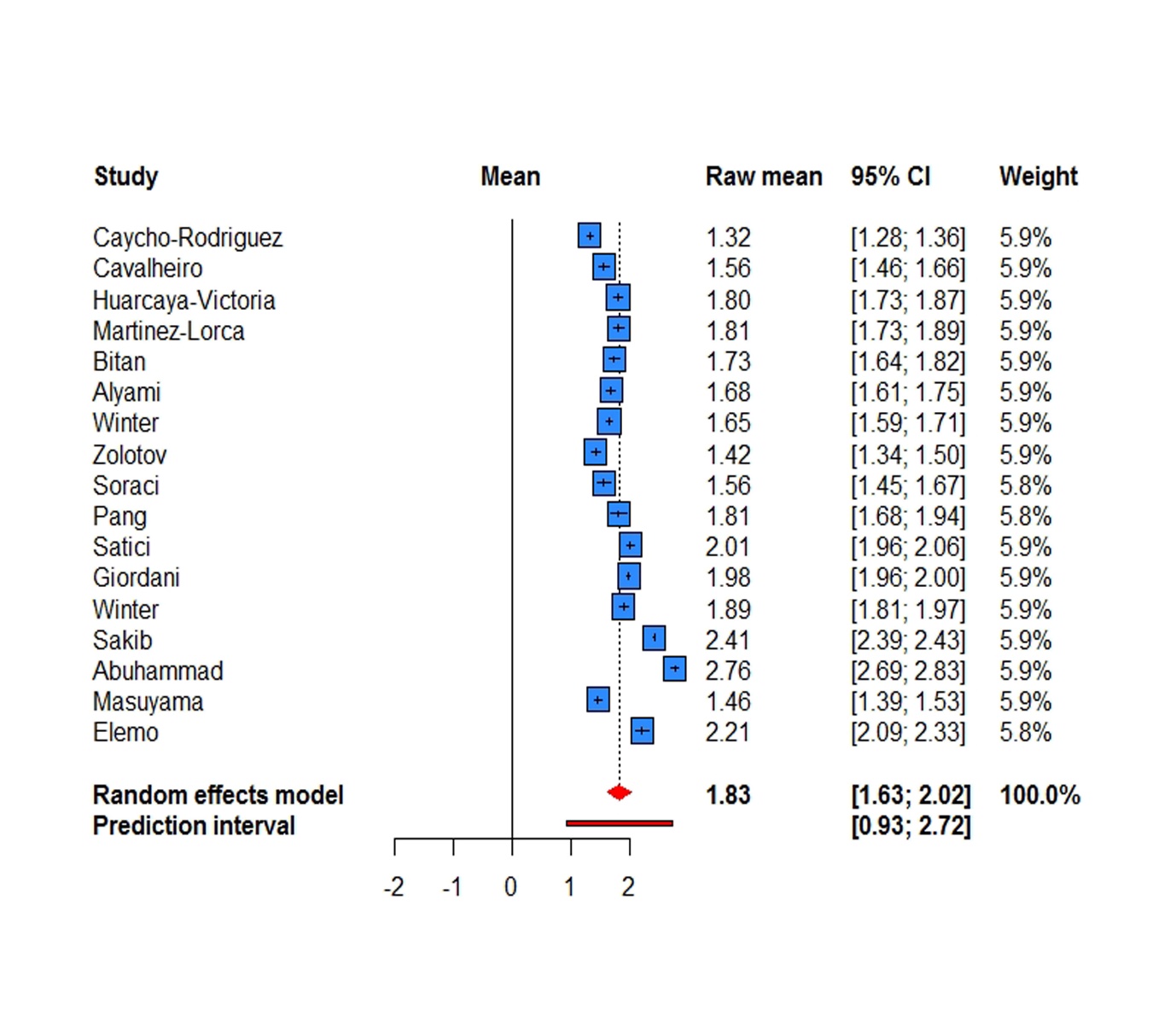


**Supplementary figure 6:** Forest plot of the fear Score mean of question 6


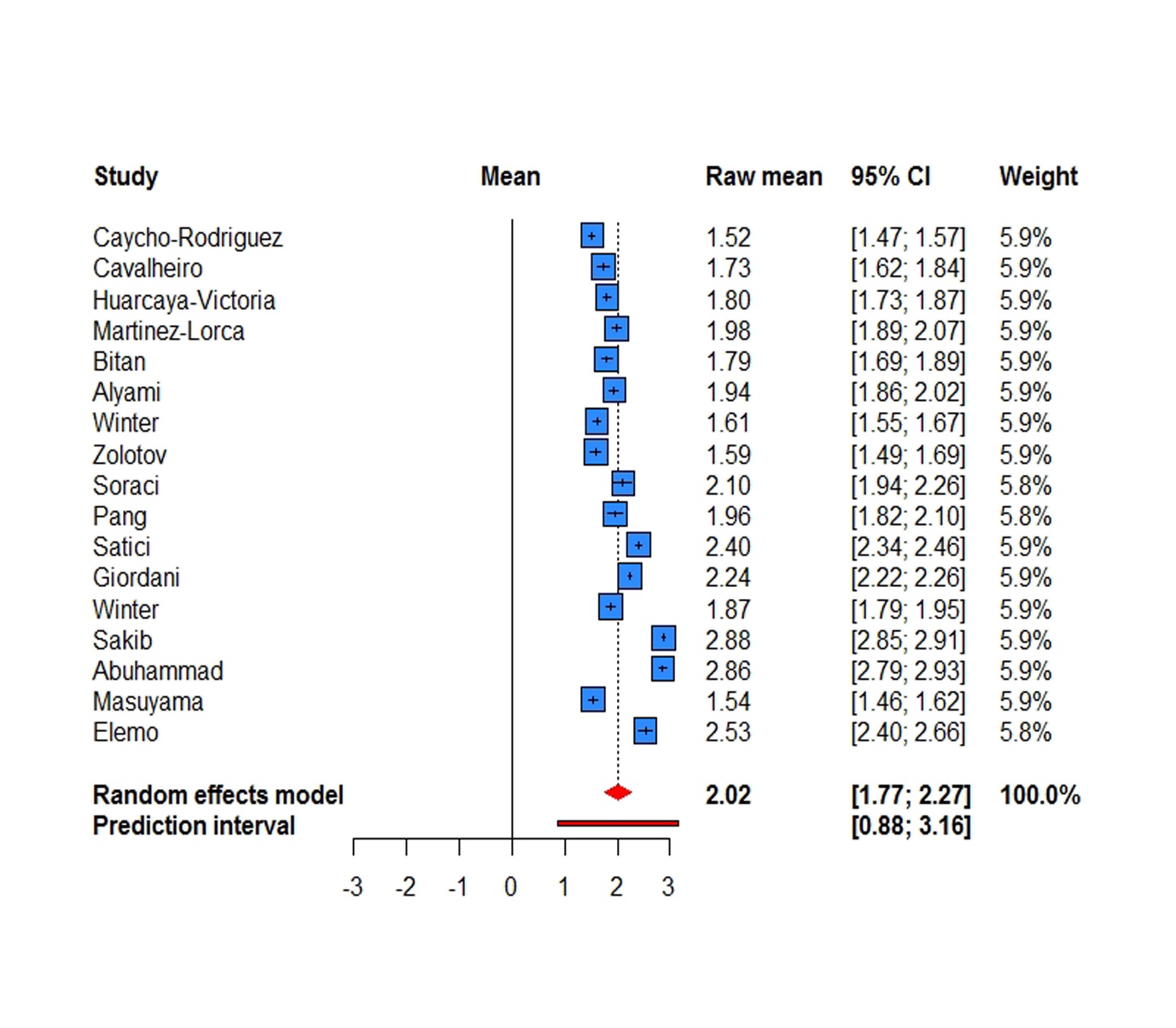


**Supplementary figure 7:** Forest plot of the fear Score mean of question 7


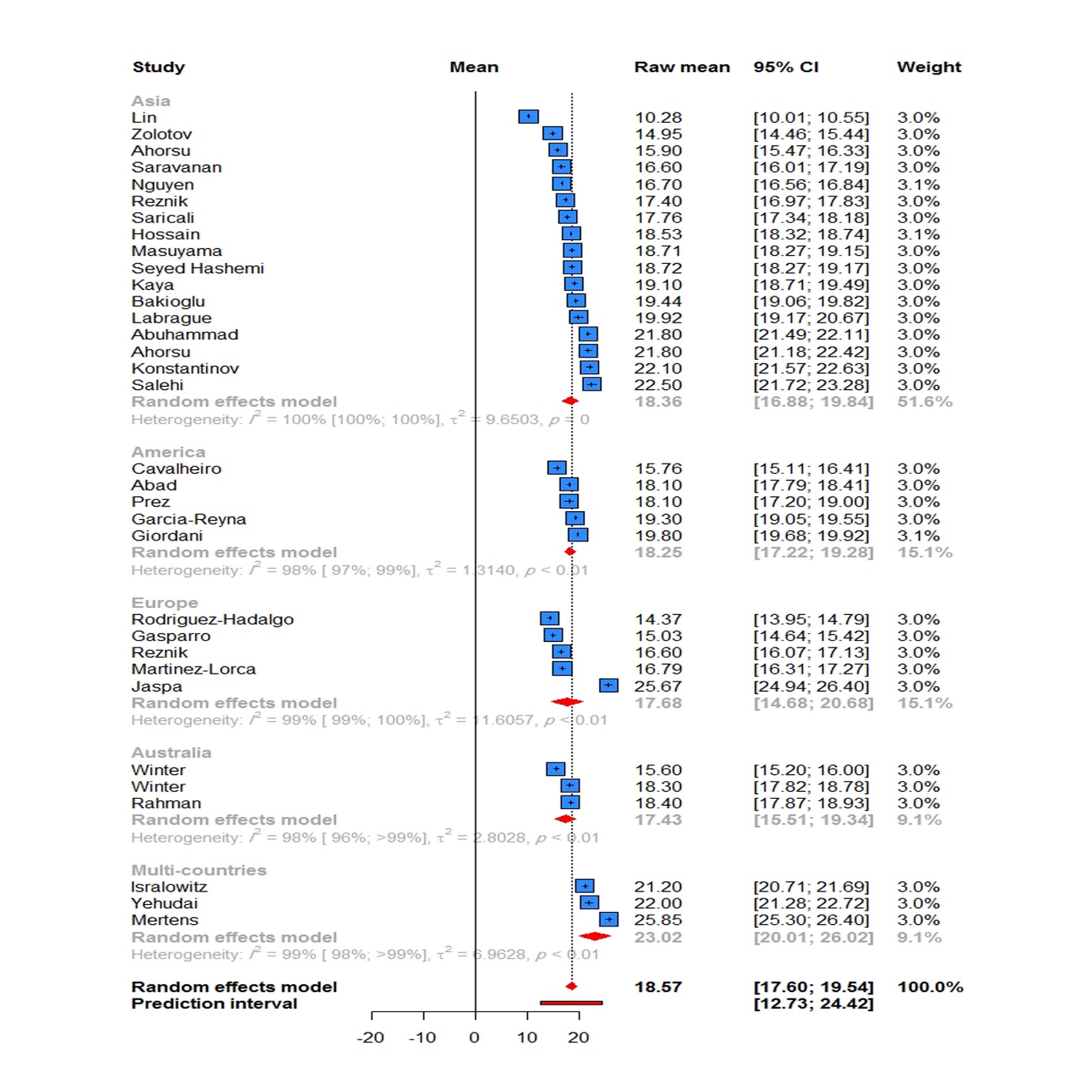


**Supplementary figure 8:** Forest plot of the total mean score of fear, according to continent


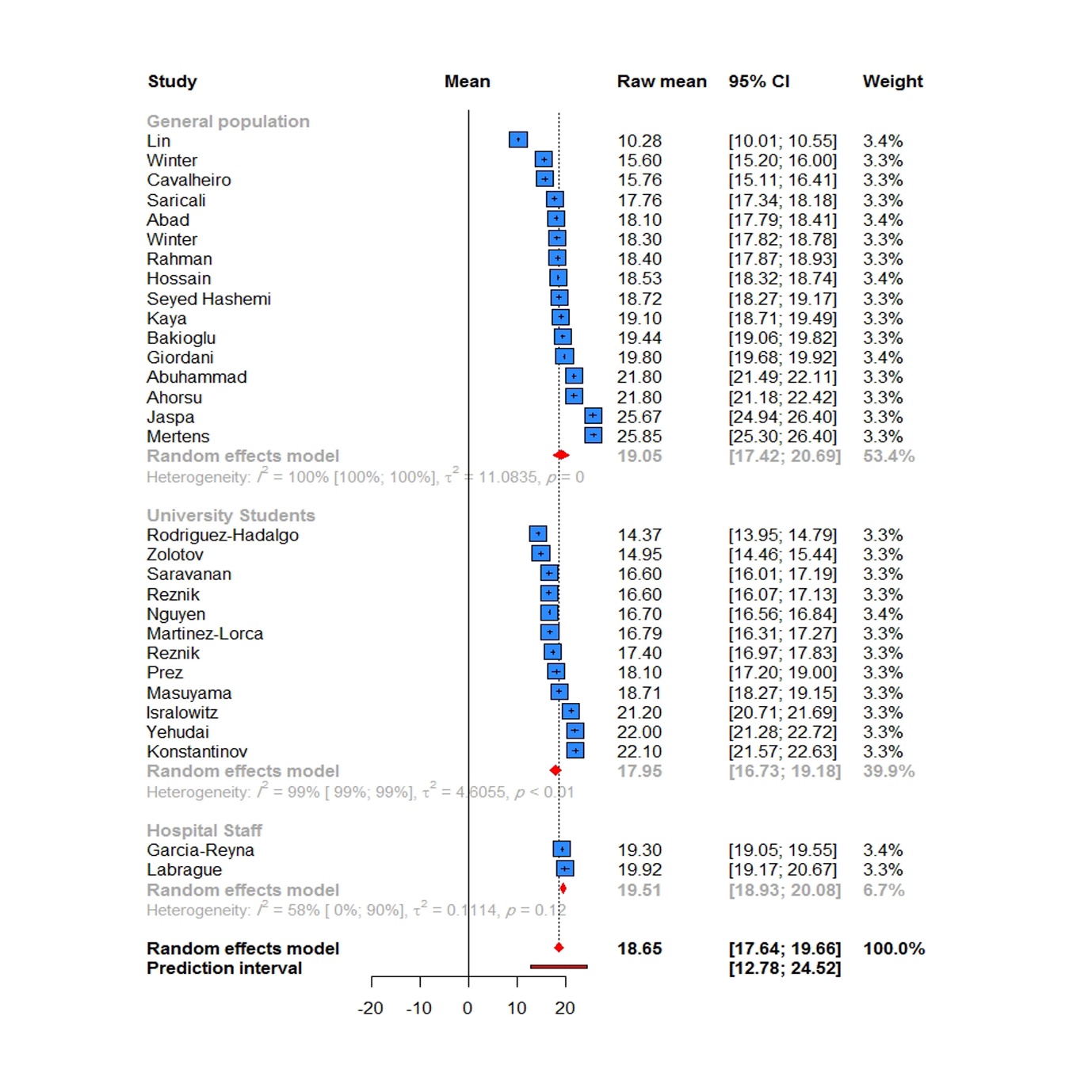


**Supplementary figure 9:** Forest plot of the total mean score of fear, according to target population


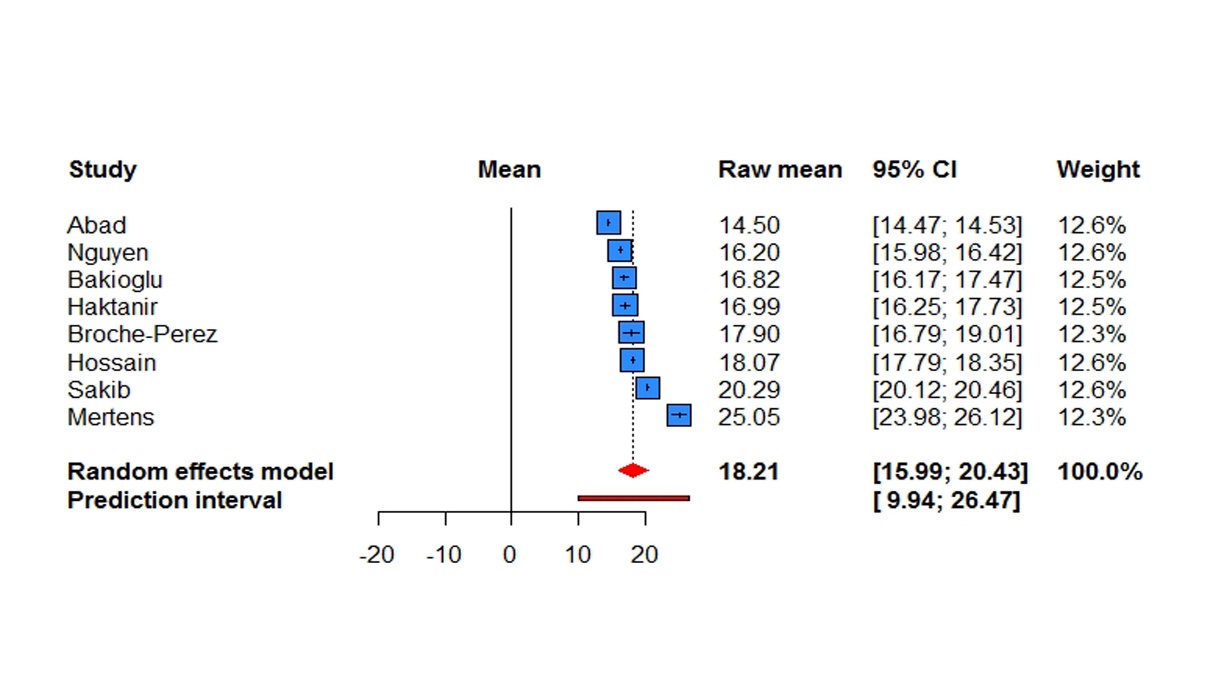


**Supplementary figure 10:** Forest plot of mean score of fear in men


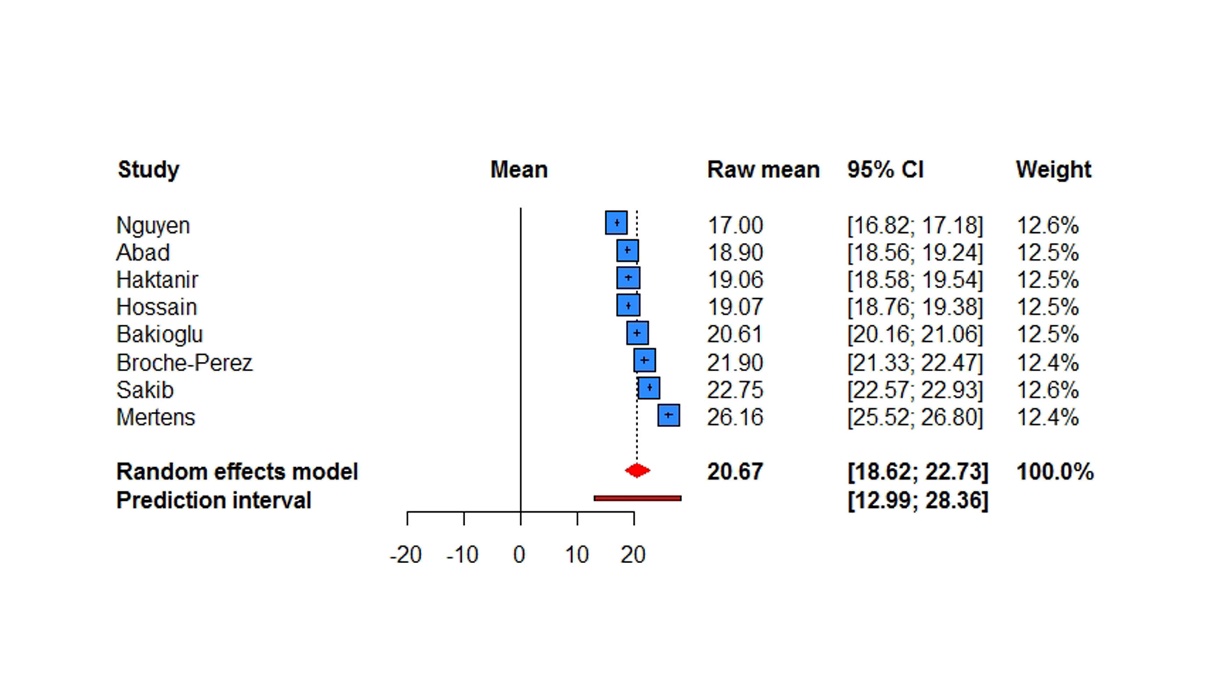


**Supplementary figure 11:** Forest plot of mean score of fear in women


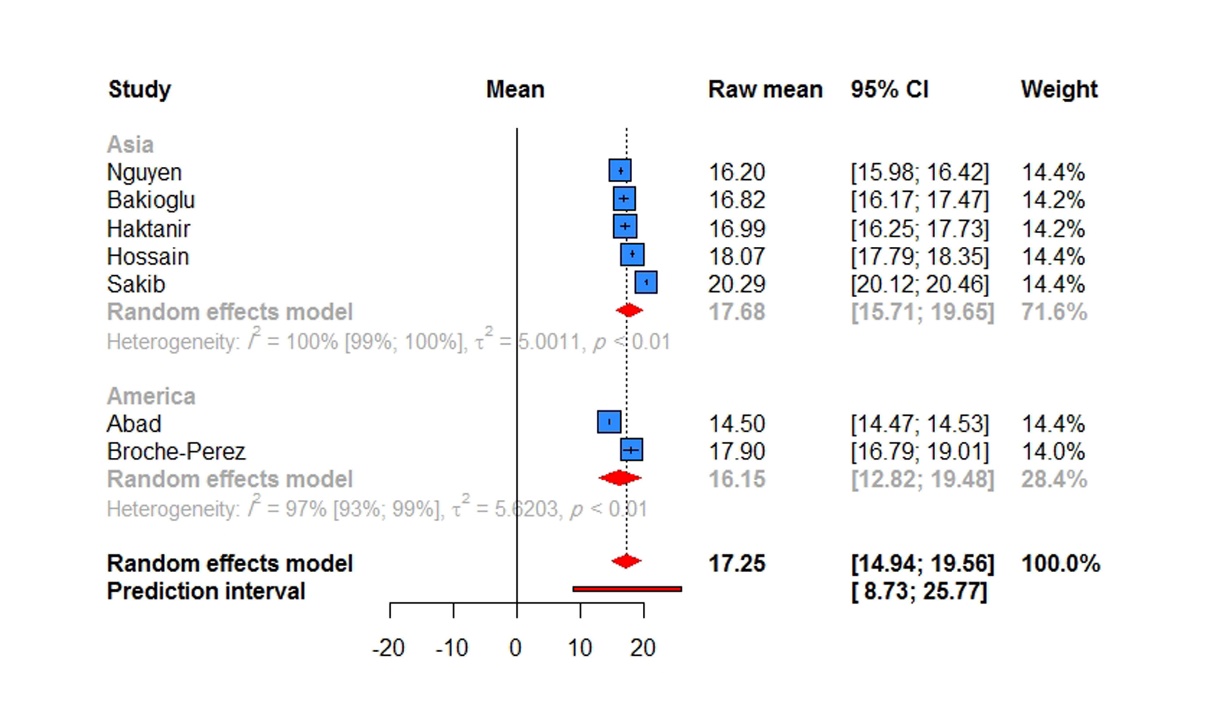


**Supplementary figure 12:** Forest plot of mean score of fear in men , according to continent


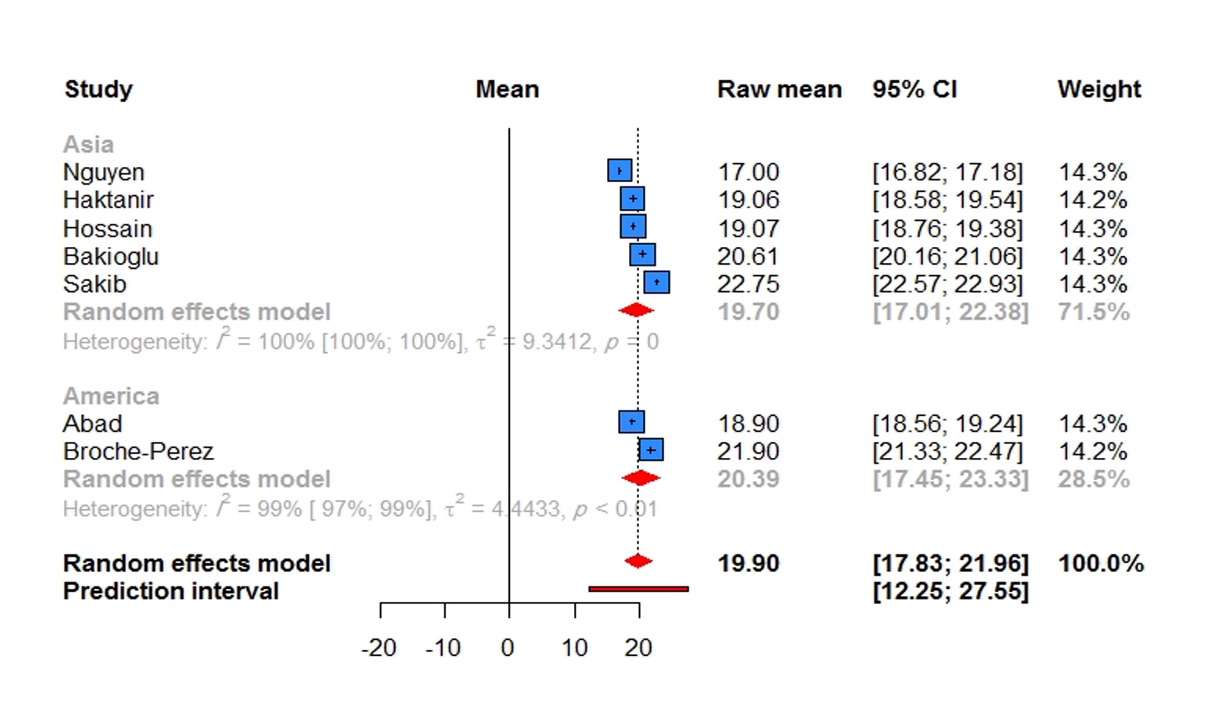


**Supplementary figure 13:** Forest plot of mean score of fear in women , according to continent


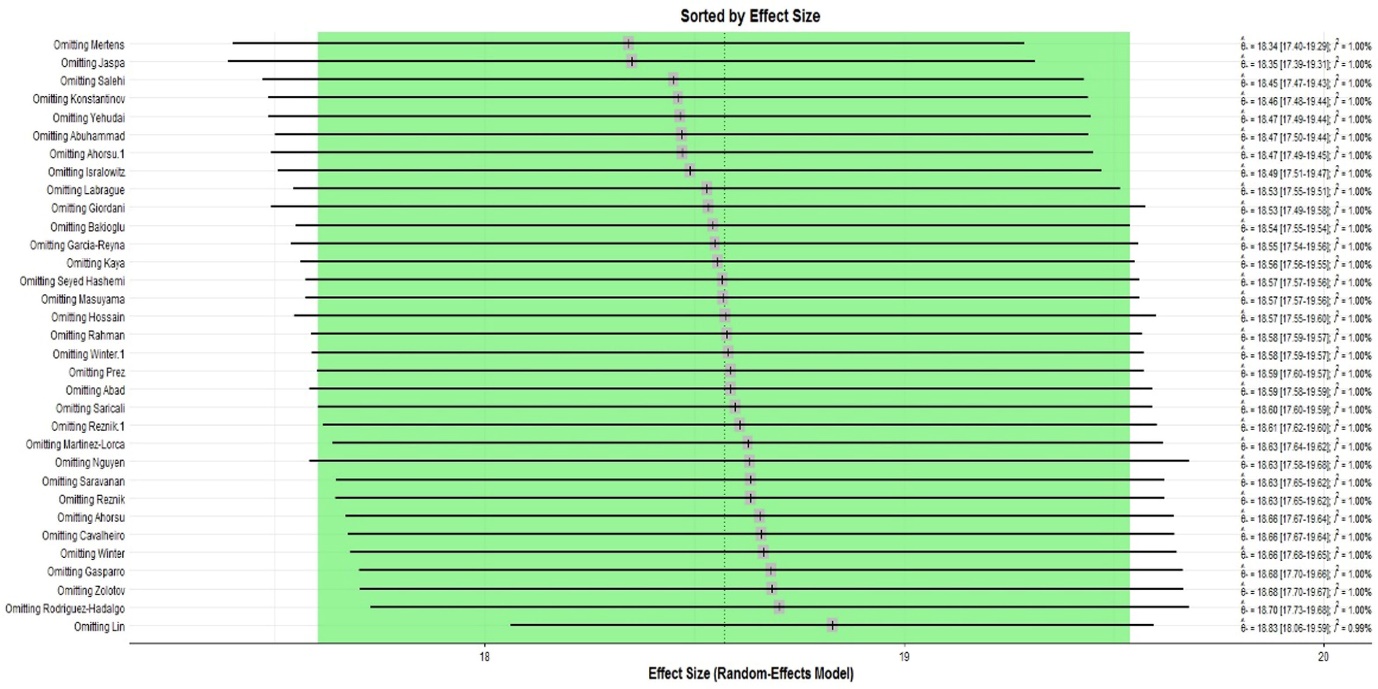


**Supplementary figure 14:** Plot of sensitivity analysis of total mean scores of fear based on leave-one-out method


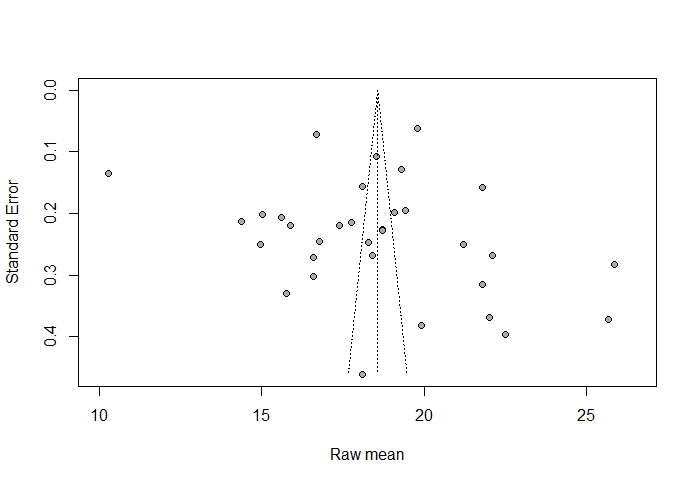


**Supplementary figure 15:** Funnel plot of publication bias based on Egger’s regression test
